# 24-hour levodopa-carbidopa intestinal gel may reduce troublesome dyskinesia in advanced Parkinson’s disease

**DOI:** 10.1038/s41531-018-0070-4

**Published:** 2018-11-20

**Authors:** Belinda Cruse, Hugo Morales-Briceño, Florence C F Chang, Neil Mahant, Ainhi D Ha, Samuel D Kim, Nigel Wolfe, Vu Kwan, David S Tsui, Jane M Griffith, Donna Galea, Victor S C Fung

**Affiliations:** 1 Movement Disorders Unit, Neurology Department, Westmead Hospital, Corner Darcy and Hawkesbury Road, Westmead, NSW 2145 Australia; 2 Sydney Medical School, University of Sydney, Sydney, Australia; 3 Department of Gastroenterology and Hepatology, Westmead Hospital, Corner Darcy and Hawkesbury Road, Westmead, NSW 2145 Australia

## Abstract

Levodopa-carbidopa intestinal gel (LCIG) is effective for the control of motor fluctuations in Parkinson’s disease (PD). The objective of this study is to report the reduction of dyskinesias after transitioning from 16 to 24-h/day LCIG infusion. From a cohort of 74 PD patients treated with LCIG for motor fluctuations, we identified 12 patients that were treated with 24-h per day infusion with the aim to control troublesome daytime dyskinesia. Clinical, demographic, dyskinesia rating scales were evaluated. Daytime dyskinesia was reduced in 75% (9/12) patients following treatment with 24-h therapy, including 7 who were compared with 16-h therapy and 2 that were transitioned from oral dopaminergic therapy to 24-h LCIG. Combining the data from all 12 subjects, troublesome dyskinesias were reduced during 24-h LCIG; UPDRS 4.1 (time spent with dyskinesias) mean change was −1.5 ± 0.75, *p* = 0.010 (Wilcoxon signed-rank test) and UPDRS 4.2 (functional impact of dyskinesias) mean change was −1.7 ± 0.90, *p* = 0.016, without changing their UPDRS part 3 “ON” scores (*p* = 0.138) or H&Y (*p* = 0.157). In 5 patients, improvement in dyskinesia occurred despite an overall increase in the total daily levodopa dose. None of the patients had worsening of dyskinesia after a median follow-up of 28 months. 24-h per day infusion of LCIG may be a useful strategy in the management of troublesome dyskinesias in PD patients with disabling dyskinesias resistant to attempts to optimise 16-hours per day therapy. We postulate that this may be due to a pharmacodynamic as opposed to pharmacokinetic mechanism.

## Motor symptoms: the benefits of round-the-clock drug delivery

Continuous 24-h intestinal administration of Parkinson’s disease (PD) drugs in a gel formulation helps control disabling dyskinesias in patients with advanced PD. Typically, patients treated with levodopa-carbidopa intestinal gel (LCIG) receive a continuous dose that is administered over 16 h. This regimen has been shown to efficiently and safely improve patients’ motor symptoms, although in some cases, daytime dyskinesias persist. Victor Fung at the University of Sydney, Australia, and colleagues examined whether 24-h LCIG infusion offers additional benefits for the treatment of dyskinesias. They found a significant decrease in the frequency and severity of daytime dyskinesia in nine out of twelve (75%) patients, and none of them experienced a worsening of dyskinesia. These results suggest that round-the-clock LCIG is a promising approach for treating troublesome dyskinesias that are resistant to 16-h therapy.

## Introduction

Continuous intra-jejunal infusion of levodopa-carbidopa intestinal gel (LCIG) is an effective advanced therapy for the treatment of motor fluctuations in idiopathic Parkinson's disease (PD),^1,2^ Currently, it is mainly utilised as a 16 h per day (16-h) continuous infusion, and is licensed for use in this fashion, however additional benefit has been reported when used as a continuous 24-hour (24-h) infusion in the treatment of severe nocturnal akinesia,^3^ day-time falls and freezing of gait (FOG),^4^ and poor sleep quality in PD.^5^

Evidence derived from randomised and open-label clinical studies support that LCIG 16-h infusion is efficacious in the treatment of levodopa induced dyskinesias (LID) despite an overall increase in mean daily levodopa dose, a benefit thought to be related to a change in levodopa pharmacokinetics with continuous drug delivery.^6,7^ The pathophysiology of LID is complex,^8^ and treatment of these disabling complications currently represents an unmet need in the treatment of patients with PD. Current treatment options for LID include amantadine, 24-h apomorphine infusion and deep brain stimulation, however these therapies are not uniformly available or suitable for all patients.

Following a single patient’s experience with improvement in daytime dyskinesia after self-initiating LCIG infusion at his day-time rate for 24 h,^4^ our centre observed similar, at times dramatic reduction in dyskinesia frequency and severity when subsequently transitioning other patients to 24-h infusion. Here we describe the experience of our movement disorder unit in the use of 24-h LCIG infusion in the treatment of dyskinesias.

## Results

The study was a retrospective analysis of all patients on 24-h LCIG therapy for whom pre-24-h and post-24-h UPDRS part 4.1 and 4.2 scores were available for analysis. Full study methods are described below. Of the 12 patients whose data were available for analysis, the indications for 24-h LCIG treatment were as follows: troublesome dyskinesias alone unresponsive to 16-h therapy (*n* = 4); troublesome dyskinesias and levodopa-unresponsive freezing of gait (*n* = 2), levodopa-unresponsive freezing of gait with non-troublesome dyskinesias (*n* = 3) or troublesome dyskinesia and nocturnal akinesia (*n* = 2), self-initiated (*n* = 1, index patient). Two patients (patients 9 and 10) were transitioned from oral levodopa therapy to 24-h therapy without a period of 16-h therapy. One previously reported patient (index patient) was included in the analysis. He had self-initiated 24-h LCIG treatment and noted a significant improvement in daytime FOG and dyskinesia.^4^ Moreover, from the same report three patients with FOG and dyskinesias were included in this study. Data for the 12 analysed patients is presented in Table 1. Due to initial intolerance of 24-h LCIG (detailed below), patient 12 had two treatment episodes for analysis (treatment 1 and treatment 2, respectively). The mean age at the time of transition to 24-h LCIG was 69 years (51-81) and mean duration of PD was 18 years (11–33). Ten patients were male (83.3%) and two of the patients had monogenic PD (*parkin* mutation patients 2 and 5). Daytime dyskinesia was reduced in 75% (9/12) patients following treatment with 24-h therapy, including 6/9 who were first treated with 16-h therapy, excluding the index case (Table 1). Combining the data from all 12 subjects, the time spent with dyskinesias (UPDRS 4.1, mean change −1.5 ± 0.75, *p* = 0.010, Wilcoxon signed-rank test) and functional impact of dyskinesias (UPDRS 4.2, mean change −1.7 ± 0.90, *p* = 0.016) were reduced during 24-h LCIG, without changing their UPDRS part 3 “ON” scores (*p* = 0.138) or H&Y (*p* = 0.157). We performed an additional analysis that included data regarding change in dyskinesia only from the 9 subjects who transitioned from 16-h to 24-h LCIG infusion, excluding the index case and also the 2 patients who initiated 24-h LCIG therapy without first undergoing 16-h therapy. The time spent with dyskinesias and the functional impact of dyskinesias was reduced during 24-h LCIG; UPDRS 4.1 mean change was −1.0 ± 1.05, *p* = 0.026 (Wilcoxon signed-rank test); UPDRS 4.2 scores −0.50 ± 1.17, *p* = 0.038, without changing their UPDRS part 3 on scores (*p* = 0.176) or H&Y (*p* = 0.317). In five patients, improvement in dyskinesia occurred despite an overall increase in the total daily levodopa dose. None of the 12 patients had a worsening of dyskinesia following use of 24-h LCIG (median follow-up, 27.5 months, range 12–54 months).

**Table 1:** Demographics and clinical evaluations of the 12 Parkinson's disease patients at baseline, 16-h and after 24-h LCIG infusion

							UPDRS			UPDRS			UPDRS			UPDRS			HY					
Pt	PD-AOO	Age at initiation 16-LCIG	Age at initiation 24-LCIG	Indication	Oral medications		Part 3			Part 4			4.1			4.2					Change in UPDRS 4.1 after 24-h LCIG	Change in UPDRS 4.2 After 24-h LCIG	Outcome	Complications
						B	16-h LCIG	24-h LCIG	B	16-h LCIG	24-h LCIG	B	16-h LCIG	24-h LCIG	B	16-h LCIG	24-h LCIG	B	16-h LCIG	24-h LCIG				
1^a^	52	62	65	Self-initiated	None	NA	9	9	11	8	2	2	2	1	3	3	0	2	2	2	−1	−1	Reduction of dyskinesias	4, 5
2^a^	30	61	63	FOG	Amantadine 300 mg/day Pramipexol 1 mg nocte	19	13	13	12	8	6	3	3	2	2	3	2	2	2	2	−1	−1	Reduction of dyskinesias	2, 4, 5, 6
3	50	63	64	Dysk	LC 100/25 mg nocte/ Amantadine 200 mg/day	35	29	24	13	9	5	4	4	1	3	2	1	2	2	2	−3	−1	Reduction of dyskinesias	None
4	44	56	57	Dysk	None	15	15	11	12	7	4	2	1	1	2	4	2	2	2	2	0	−3	Reduction of dyskinesias	4, 5
5	42	68	71	Dysk	LC CR 200/50 mg nocte	26	28	31	10	11	6	3	3	2	3	4	2	2	2	2	−1	−2	Reduction of dyskinesias	1, 2, 4, 5
6	59	69	70	Nocturnal akinesia and daytime Dysk	None	49	10	10	9	4	3	1	1	1	0	0	0	2	2	1	0	0	No improvement of dyskinesias	2, 4
7^a^	66	81	81	FOG	None	29	37	23	7	7	6	0	0	0	0	0	0	2	2	1	0	0	Improvement of FOG- No dyskinesias	4
8	65	78	80	Dysk	LC CR 200/50 mg nocte	31	17	18	11	9	6	3	3	2	3	3	2	2	2	2	−1	−1	Reduction of dyskinesias	4
9^a^	61	n/a	81	FOG and Dysk	Pramipexol 1 mg nocte	52	65	40	9	8	6	3	3	1	0	0	0	3	3	3	−2	0	Reduction of dyskinesias	1, 4
10	39	n/a	67	FOG and Dysk	None	46	42	44	13	11	12	2	2	1	2	2	1	3	2	2	−1	−1	Reduction of dyskinesias	3, 4, 5, 6
11	40	50	51	Nocturnal akinesia and daytime Dysk	None	17	13	7	10	5	7	2	3	1	1	1	1	1	1	1	−2	0	Reduction of dyskinesias	5
12 (treatment 1)	63	n/a	74	FOG	None	24	18	18	8	6	5	1	1	1	1	1	1	2	2	2	0	0	No improvement of dyskinesias/ FOG improved but returned to 16-h due to hallucinations	1, 4, 5
12 (treatment 2)		74	77	FOG and Dysk	None	22	23	24	11	11	11	2	2	2	4	4	4	3	3	3	0	0	No improvement of dyskinesias/ FOG improved	1

*PD-AOO* Parkinson’s disease age of onset, *B* baseline, *UPDRS* Unified Parkinson’s Disease Rating scale, *HY* Hoehn & Yahr, *Dysk* dyskinesia, *FOG* freezing of gait, *LC* levodopa/carbidopa, *LC CR* levodopa/carbidopa controlled release, *n/a* not applicable

^a^Patients previously reported: Ref. ^4^

1-Worsening of previous nocturnal hallucinations; 2-Non-disabling neuropathy with 16-h LCIG; 3-Asymptomatic *de novo* neuropathy ; 4-Vitamin B6 deficiency ; 5-Vitamin B 12 deficiency; 6-Hyperhomocysteinaemia

With regards to the infusion regimen in responders, seven patients had an overall increase in the 24-h levodopa equivalent, and two (patients 4 and 8) had an overall decrease in the 24-h levodopa equivalent (Table 2). Overall, the mean change in daily levodopa dose was not significantly increased following the transition to 24-h LCIG (30.9 ± 46.4 %, *p* = 0.074) and at the latest follow up (34.6 ± 50.7%, *p* = 0.114), compared to 16-h LCIG. In the 5 patients with dyskinesia improvement and overall increased 24-h levodopa equivalent, this occurred despite an increase in the daytime LCIG continuous rate (CR) in two cases (patients 3 and 11), and unchanged or reduced LCIG daytime CR in 3 cases (patients 1, 2, and 5) (Table 2).

**Table 2:** Infusion rates and daily levodopa equivalents during 16-h and after transition to 24-h LCIG infusion

Patient	Daytime CR on 16-h therapy	Total daily L-DOPA equivalent on 16-h infusion (or oral therapy if direct to 24h Rx)	Daytime CR on 24-h infusion at time of follow-up assessment	Night CR on 24-h infusion at time of follow-up assessment	Total daily L-DOPA equivalent on 24-h infusion (after initial titration)	Change in 24 h levodopa dose (%)	Most recent day rate	Most recent night rate	Most recent follow up 24-h L-DOPA equivalent	Change in 24 h L-DOPA dose since 24-h therapy initiation and latest follow-up (%)	Follow-up duration ON 24-h LCIG (months)
1	4.1	1572	3.9	3	1734	110	5.1	4.1	2260	130%	57
2	1.5	570	1.5	1.1	656	115	2.2	1.5	944	127%	54
3	3.8	1394	3.9	1.7	1550	111	4.5	4.3	2158	139%	49
4	6.7	2364	2	2	960	41	4.5	3.2	2080	217%	43
5	6.1	2292	6.1	4.2	2744	120	4.5	2.6	1856	68%	33
6	7	2550	7.3	5.1	3292	129	8	6	3664	115%	28
7	3	1270	4.3	3	1936	152	5.4	3.5	2368	172%	27
8	5.1	2102	3.5	3.5	1300	62	4.1	2.7	1764	135%	25
9	n/a	1380	5.4	3.2	1604	116	5.5	3.2	2452	152%	23
10	n/a	1275	5.7	4.7	2656	208	6.1	4.9	2796	135%	20
11	2.6	966	3	2.1	1390	144	3.5	2.5	1562	136%	15
12 (treatment 1)	n/a	1125	5.1	3.5	2192	195	2.2	0.5	716	33%	48
12 (treatment 2)	2.8	784	2.8	0.5	1016	130	2.2	0.5	716	91%	12

Three patients (patients 6, 7, 12) had no reduction in dyskinesias. One of these patients (patient 12) was transitioned to 24-h LCIG for the treatment of severe FOG and dyskinesias, however could not tolerate 24-h infusion due to worsening of hallucinations and agitation (treatment 1 in Table 1), and was transitioned back to 16-h therapy after 2 months. This patient had pre-existing cognitive decline and hallucinations. After 6 months neurocognitive function returned to baseline. The patient was re-trialled on 24-h therapy (treatment 2) eleven months later for the treatment of disabling and distressing dyskinesia without complication, but without benefit with respect to dyskinesia. The remaining 2 patients transitioned to 24-h therapy for FOG or nocturnal akinesia with non-troublesome dyskinesias, had no change in dyskinesia scores despite an improvement of FOG or nocturnal akinesia.

Side effects are summarised in Table 1. In addition to patient 12, two other patients had worsening of nocturnal hallucinations (patients 5 and 9) which improved after down-titration of the night-time LCIG CR. At commencement of 24-h LCIG therapy, 3 patients (27%) had pre-existing, non-disabling peripheral neuropathy that was managed with surveillance nerve conduction studies and clinical follow-up, and none developed worsening of peripheral neuropathy. One patient developed asymptomatic peripheral neuropathy within 6 months after commencing de novo 24-h LCIG therapy and this remained stable over 18 months of follow-up. Ten of the 12 patients (83%) developed vitamin B6 deficiency requiring supplementation, 7 (58%) also developed vitamin B12 deficiency during therapy (either 16-h or 24-h). Two patients had hyperhomocysteinaemia. No patient elected to return to 16-h therapy because of difficulty managing the pump, or technical difficulties with the PEG-J tube.

## Discussion

We report a reduction in daytime dyskinesia in 9 out of 12 patients following treatment with 24-h LCIG therapy. This improvement was observed despite an increase in total daily levodopa dose in 5 of the 7 patients that had a trial of 16-h LCIG. In 2 of these 7 patients, the daytime continuous rate did not change, and in a further 2 the daytime rate was increased. None of the 12 patients had a worsening of daytime dyskinesia following the transition to 24-h LCIG. We are not able to conclude whether this improvement represents a reduction in peak-dose, biphasic dyskinesias, or a combination of these in individuals in this study, and this would be important to evaluate in future studies. Notably, the ‘ON’-UPDRS part 3 scores remained similar following transition to 24-h infusion, and the daytime rate was the same or lower in 5/7 responders transitioned from 16-h infusion, which may suggest an improvement in peak-dose dyskinesias, however again this requires further evaluation before conclusions can be drawn. The remaining 2 patients that were transitioned from oral levodopa to 24-h LCIG also improved dyskinesia and motor scores; however it is possible that they would also have improved with a 16-h LCIG trial.

The pathophysiology of levodopa induced dyskinesias (LID) is a complex phenomenon comprising a combination of nigrostriatal degeneration, pulsatile dopaminergic stimulation and synaptic remodelling.^8^ LCIG has the capacity to influence both pharmacokinetic and pharmacodynamic factors, but until now pharmacokinetic factors have been thought to be the dominant mechanism.^6^ This is supported by the effectiveness of 16-h LCIG, where improvements in dyskinesia can be observed rapidly within hours to days of commencing therapy.^6^ However, evidence derived from clinical studies suggests that pharmacodynamic factors may also play a role in LID.^9–12^ The pharmacodynamic effects of LCIG from studies of 24-h intravenous levodopa infusion demonstrated an increase of the threshold for LID with a reduction in the severity of dyskinesia, independent of the antiparkinsonian effect of levodopa.^5,11–13^ We also observed a reduction in dyskinesias without loss of antiparkinsonian effect over the course of LCIG therapy despite the increment of total daily levodopa dose. The improvement in dyskinesia observed in some patients with 24-h LCIG, which could not be controlled with 16-h LCIG at the same or lower continuous rate, suggests that this improvement is due to pharmacodynamic processes that may rely on steady, continuous levodopa delivery to the brain. Due to the limitations in our study, we are unable to draw conclusions regarding potential mechanisms of our observations; this should be a focus of future research in this area.

In this cohort, there was a wide range in night-time LCIG rates at the time of follow-up (0.5–5.1 ml/h; 43–100% of day-rate, median 69%). Our approach when transitioning to 24-h LCIG is to commence the night-time rate at approximately 50% that of the day rate. If tolerated, slow up-titration (0.1 ml/h of night-time rate) in 1–4 weeks intervals, is then attempted, until therapeutic benefit and/or side effects are encountered. Based on this study it is not possible to determine the minimal night-time LCIG rate at which dyskinesia may be improved, however this is an important aspect of management which should be evaluated in prospective trials. It is interesting to note that at the time of follow-up, the night time rate varied from 43% of the day rate (patient 3) to 100% (patient 4) of the concurrent day rate among the responders (Table 2).

The side effects observed in our patients are similar to previously reported studies with 16-h LCIG infusion.^14^ Overall the treatment was well-tolerated as only one patient needed to revert to 16-h LCIG (patient 12, due to worsening hallucinations). Two other patients had an increase in hallucinations, effectively managed by the reduction in night-time LCIG rate or observation only without losing the anti-dyskinetic effect. One patient developed a transient delirium in the context of urinary tract infection and inadvertent exposure to daytime LCIG rate across the night (patient 9). Although none of the patients had de novo symptomatic neuropathy nor exacerbation of preexisting neuropathy, many of the patients developed vitamin B6 or vitamin B12 deficiencies. Development of neuropathy with LCIG has been related to levodopa total daily dose, deficiency of vitamin B12 and B6 and hyperhomocysteinaemia.^15^ Our standard practice involves close monitoring (6 monthly or more frequent if deficiency identified) of serum vitamin B6/12/red cell folate and homocysteine in these patients, vitamin replacement as needed, and surveillance examination and NCS/EMG (6–12 monthly).^16^

There are several limitations to the study, the most significant being the retrospective nature of the analysis, the lack of blinded evaluation for dyskinesias and motor scores, and the lack of a comparator group. Seven of the 9 patients who were transitioned to 24-h therapy for the treatment of troublesome dyskinesia however had trialled 16-h LCIG, therefore comparison of 24-h vs. 16-h LCIG therapy is made possible in this group.

In conclusion, this retrospective study has demonstrated a reduction in troublesome dyskinesia duration and its functional impact following the use of 24-h LCIG therapy in some patients. We acknowledge the limitations of the present study and larger prospective, controlled studies are needed to confirm these observations. However if confirmed, 24-h LCIG may offer a novel approach to the treatment of troublesome dyskinesias which persist despite an adequate trial of 16-h therapy. We hypothesise that the decrease in LID despite increased levodopa dose and rate in some of our responders suggests the possibility of a pharmacodynamic mechanism in at least some patients.

## Methods

We conducted a retrospective analysis of data collected on all PD patients initiated with 24-h LCIG within our centre from September 2012 to August 2016. Patient demographics, clinical characteristics, dyskinesia severity and incidence were reviewed, before and after the transition to 24-h therapy using UPDRS part III, part IV total scores and sub-scores 4.1 (time spent with dyskinesias) and 4.2 (functional impact of dyskinesias). All rating scales and evaluations were performed at baseline and at 6 months after starting 16-h and 24-h LCIG. Total daily (24-hour) levodopa dose, and where applicable daytime and night-time CR of LCIG before and after the transition to 24-h therapy, were analysed. The total daily dose of LCIG at most recent follow up was also calculated and compared with that at initiation of 24-h therapy. Levodopa equivalent dose was calculated for all dopaminergic therapy using established conversion factors.^17^ Side effects were documented, specifically the incidence of hallucinations, psychosis, impulse control disorder, dopamine dysregulation syndrome and peripheral neuropathy. All the participants provided written informed consent to take part in the study.

From a total of 74 patients on treatment with LCIG at the time of the study, 34 were on treatment with 24-h LCIG. We selected for the current study those patients on 24-h LCIG who had clear documentation of dyskinesias at the time of their initiation of 24-h treatment for whom sufficient documentation of pre- and post-24-h treatment was available for analysis (*n* = 12). The 22/34 patients excluded from analysis either were converted to 24-h for reasons other than dyskinesias e.g., nocturnal akinesia, levodopa-unresponsive FOG without documented dyskinesia, or clinical information about their pre- and post-24-h LCIG dyskinesias was incomplete. The study was approved by the Western Sydney Local Health District Human Research Ethics Committee. We used SPSS 23.0 software and Wilcoxon signed rank test to compare 16-h and 24-h LCIG Hoehn and Yahr, UPDRS part 3, 4.1 and 4.2 sub-scores and daily levodopa dose with *p* < 0.05 set as significant. The authors declare that all the data supporting the findings of this study is available within the paper.

## Data Availability

All data generated or analysed during this study are included in this published article and its supplementary information files.
